# Glycosylation as a strategic mechanism for measles virus and mumps virus immune evasion

**DOI:** 10.3389/fimmu.2025.1716829

**Published:** 2025-12-10

**Authors:** Catalina Galvan, Inna G. Ovsyannikova, Richard B. Kennedy

**Affiliations:** Mayo Clinic Vaccine Research Group, Mayo Clinic, Rochester, MN, United States

**Keywords:** glycosylation, measles, mumps, paramyxovirus, MMR vaccine

## Abstract

Glycosylation of viral surface proteins by host cell factors is one strategy paramyxoviruses employ to evade the host’s immune system during infection. Viral glycosylation thus has the potential for innate and adaptive immune modulation. However, an adequate assessment of the effects glycosylation has on immune recognition and response for two important paramyxoviruses, Measles virus (MeV) and Mumps virus (MuV), is lacking. This review aims to provide a comparison of epitope-site sequence changes in the surface glycoproteins MeV-H, MeV-F, MuV-HN, and MuV-F across different wild type and vaccine strains of measles and mumps. Such changes may alter glycosylation patterns at antigenic sites, thus altering the virus’ efficiency to induce an immune response as well. Further investigation of measles and mumps viral glycosylation studies will aid the development of specific therapeutics that modulate viral glycosylation during immune diseases, viral infections, and oncolytic treatments. Moreover, determining how glycosylation affects measles and mumps immune responses may pave the way for the development of novel vaccine strains for the improved immunogenicity and immune durability of measles and mumps vaccines.

## 1 Introduction

Measles and mumps are re-emerging worldwide, and their outbreaks are predicted to remain prevalent (1–8). Among those vaccinated against mumps, significant waning immunity to mumps has been reported in recipients, with protection estimated to only last for 10–15 years after receiving the MMR vaccine (1, 2, 9). Interestingly, waning immunity is substantially greater to mumps than to measles after MMR vaccination for reasons currently unknown (9). Mumps outbreaks have increased in recent decades within vaccinated populations with reported waning immunity (5–11). Waning immunity could thus potentially result in larger outbreaks of mumps as compared to measles in the future (1, 2, 9). This marks a growing need to control measles and mumps outbreaks in order to forestall the re-introduction and re-establishment of endemicity in geographic areas that have achieved local elimination to minimize or prevent epidemics and pandemics in the future (1–4, 10–13). Vaccination with the live-attenuated measles, mumps, rubella (MMR) vaccine (see **Table 1** for a list of abbreviations), which induces protective immunity against measles, mumps, and rubella, is of yet the most effective approach to outbreak prevention for these viral diseases (9, 14, 15). Furthermore, developing antiviral therapies may enhance the treatment of severe cases (16, 17). Additionally, vaccines can be improved to be able to offer as viable options for immunocompromised individuals and others who have contraindications to the current live-virus vaccines. Therefore, a need for novel antiviral treatments and improved vaccines for measles and mumps are necessary to stop measles and mumps outbreaks from growing out of hand (16–19).

**Table 1:** In-text abbreviations.

Abbreviation	Definition
MMR	Measles, mumps, rubella
MeV	Measles virus
MuV	Mumps virus
HeV	Hendra virus
NiV	Nipah virus
N-	Asparagine linked
LLO	Lipid-linked oligosaccharide donor
ER	Endoplasmic reticulum
GalNAc	N-acetylgalactosamine
NeuAc	N-acetylneuraminic acid
Gal	Galactose
O-	Serine/Threonine linked
mAbs	Monoclonal antibodies
LayV	Langya henipavirus
MojV	Mojiang henipavirus
RBS	Receptor binding site
HNE	Hemagglutinating and noose epitope
SLeX	Sialyl Lewis X
blastp	Protein-to-protein BLAST
JL-2	Jerryl-Lynn 2
JL-5	Jerryl-Lynn 5
Abs	Antibodies
PRR	Pattern recognition receptor
SAMP	Self-associated molecular pattern
PAMP	Pathogen-associated molecular pattern
TLR	Toll-like receptor
CLR	c-lectin receptor
IFN1	Type I interferon
MAC	Membrane attack complex
NF-κB	Nuclear factor kappaB
STAT1	Signal transducer and activator of transcription 1
STAT3	Signal transducer and activator of transcription 3
SARS-CoV-2	Severe acute respiratory syndrome coronavirus 2
HIV	Human Immunodeficiency virus
AIDS	Acquired Immunodeficiency syndrome

One way to develop effective antivirals and next-generation vaccines is to harness cellular mechanisms that counter the immune evasion strategies used by measles virus (MeV) and mumps virus (MuV) (20–27). To do so, it is imperative to confirm that immune evasion strategies identified in other paramyxoviruses are utilized by measles and mumps (20–28).

Hendra virus (HeV) and Nipah virus (NiV), members of the Paramyxovirus family like MeV and MuV, also result in serious respiratory and neurological illnesses, including pneumonia and encephalitis (1, 14, 28–30). Thus, they too pose a significant public health concern (28–30). Research that aids in identifying immune evasion strategies in paramyxoviruses will inform novel methods to study paramyxovirus immunomodulation. New methodologies that can be used to study the immunomodulatory effects of different viral glycosylation patterns across different paramyxoviruses will be a useful tool in engineering, not only antivirals and next generation vaccines for measles and mumps, but antivirals and vaccines that can prevent other human paramyxoviruses as well (3, 4, 12, 16, 17, 31).

Glycosylation is one immune evasion strategy HeV and NiV use to hide immunogenic viral epitopes on their surface (32–34). This will be termed throughout this review as glycan-shielding. For glycan-shielding to occur successfully, the virus must become glycosylated at specific epitope sites so that the attached glycan can prevent Abs from binding and neutralizing the virus (35, 36). For both HeV and NiV, it has been demonstrated that glycosylation aids viral entry and spread within the host (32–34). Moreover, glycosylation prevents HeV and NiV from being neutralized by virus-specific Abs (32–34, 37). Despite what is known about glycan-shielding in HeV and NiV, whether or not it occurs in MeV and MuV remains an open question. Furthermore, the downstream immunomodulatory effects glycan-shielding has on host cells during paramyxovirus infection in general is an area that remains unstudied.

This review will highlight differences in amino acid sequences of MeV and MuV surface glycoproteins MeV-H, MeV-F, MuV-HN, and MuV-F that may alter viral epitope glycosylation. Additionally, this review will emphasize that a greater understanding of how MeV and MuV potentially take advantage of glycan-shielding can lead to the development of improved treatment options. These may include the creation of novel viral vaccine strains that possess specific glycosylation sites in order to yield a specific immune response, or drugs that interfere with or alter glycosylation in ways that enhance immune recognition or diminish viral infectivity.

## 2 Paramyxovirus glycosylation

Two main mechanisms of glycosylation, which are conserved across prokaryotes and eukaryotes, are N-linked glycosylation and O-linked glycosylation (38–40). MeV and MuV become N-linked and O-linked glycosylated within the endoplasmic reticulum (ER) and Golgi apparatus of host cells during infection by taking advantage of the host’s secretory pathways (41–49). This implies that glycosylation of MeV and MuV may not only impact envelope fusion but viral assembly and intracellular transport as well. This hypothesis has yet to be studied for MeV and MuV but has previously been proven true for other enveloped viruses in the Picornaviridae, Coronaviridae, Flaviviridae, Poxviridae, Parvoviridae, and Herpesviridae families (32). MeV-H and MeV-F surface glycoproteins are modified by host enzymes through N-linked glycosylation as they move through the endoplasmic reticulum and Golgi, a process essential for correct protein folding, processing, and placement on the cell surface (50, 51). MuV proteins interact with host glycosyltransferases in the ER and Golgi to become glycosylated (52, 53). Some of the sites that the surface glycoproteins MeV-H, MeV-F, MuV-HN, and MuV-F in which MeV and MuV become N- and O-linked glycosylated have been identified (41–44, 46–48). However, compared to other paramyxoviruses, such as NiV and HeV, it is far less understood what effects MeV and MuV glycosylation have on virus infectivity, fusogenicity, and spread (32–36). This demonstrates a need for future studies to more comprehensively characterize potential glycosylation sites and to analyze the effects of glycosylation on immune responses to MeV and MuV.

For example, glycosylation aids NiV in viral entry, spread, and evasion of host cell Abs (32, 37). One study revealed that N-glycan removal of the F protein in NiV resulted in an increased sensitivity to neutralization by host Abs (see **Table 2**) (37). Furthermore, it was discovered that two divergent strains of NiV that have different glycosylation sites from NiV, Langya henipavirus (LayV) and Mojave virus (MojV), were unable to be neutralized by NiV-specific polyclonal sera for NiV-F raised in mice (55). Glycan shielding was further proven to be the reason behind evasion of NiV-specific Abs when the identification of a glycan at the DIII apex was found to be able to shield both LayV and MojV from mAbs such as mAb66 (55). In addition, mutating out known O-glycan locations in the HeV and NiV G proteins negatively affected both viruses in their ability for host cell entry and spread (55).

**Table 2:** Mutations to NiV and HeV glycosylation sites.

Viral protein	Glycosylation site change	Reference
HeV stalk domain (aa103-137)	Serine/Threonine residue ➔ Alanine	Bradel-Tretheway, B.G. et al, 2015 (54)
NiV F_2_ subunit (F1, F2, F3)	Asparagine ➔ glutamine at position 1 of the glycosylation sequon N^1^X^2^(S/T)^3^	Aguilar, H. C. et al, 2006 (34)
NiV F_1_ subunit (F4, F5)		

HeV is also able to escape neutralization by host cell antibodies by glycosylation (32, 54). In one study, an antibody neutralization assay comparing WT and mutant HeV G virions demonstrated that most N-glycans in HeV G protected the virus from antibody neutralization (see **Table 2**) (54). One example of a glycan HeV uses to escape neutralization is the HeV G N-glycan at position G5 (residue 417) (54). Since G5 is located near a major neutralizing epitope site, the glycosylation of this site prevents Abs from binding to the epitope, thereby interfering with the neutralizing activity of that antibody (54).

**Figure 1:**
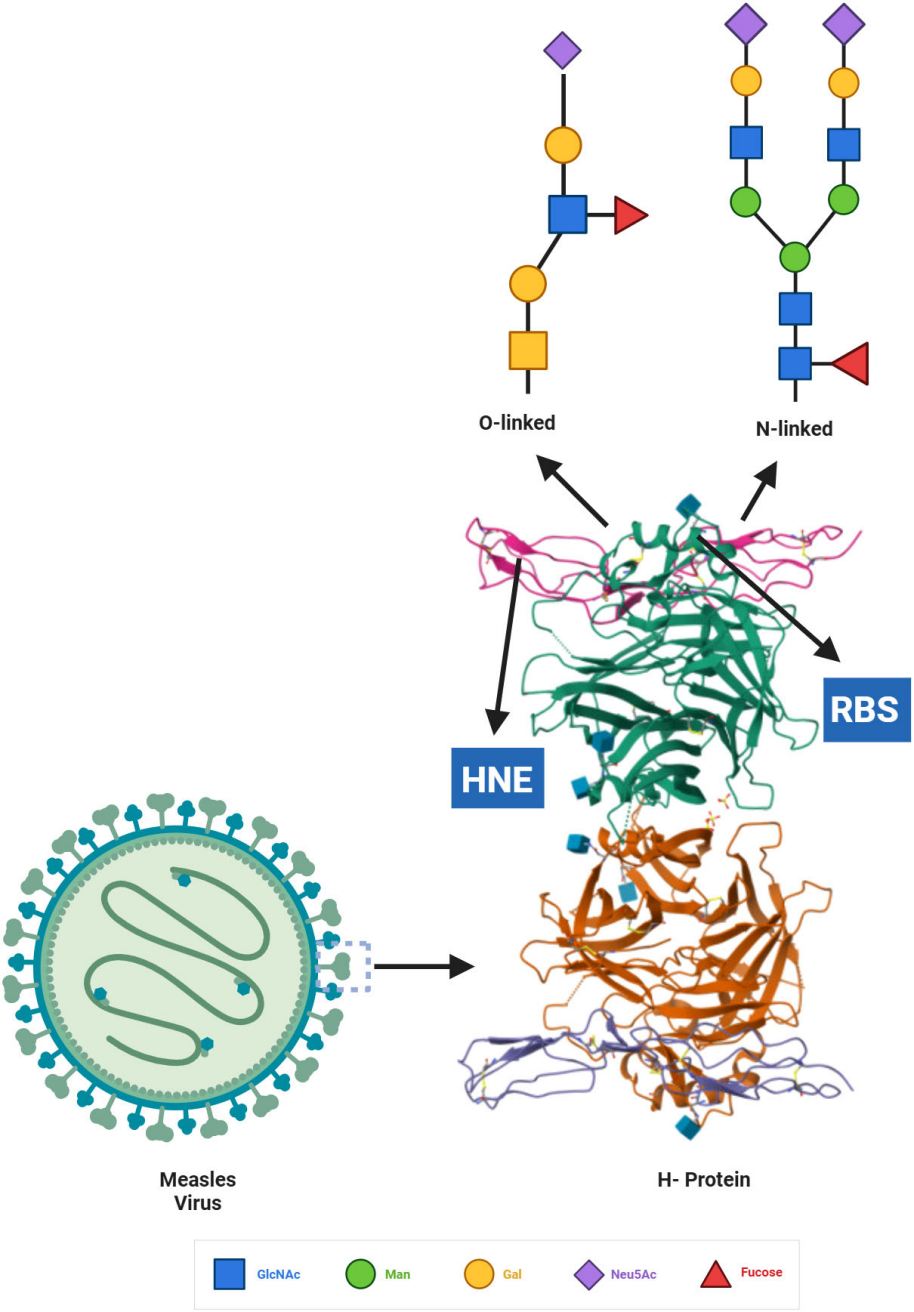
Glycosylated measles H protein. N-linked and O-linked glycans bind to the head domain of the surface glycoprotein MeV-H. Within the head domain are the virus-specific receptor binding sites (RBS) and hemagglutinin noose epitope (HNE). MuV-H structure used in the figure was obtained from updated resources for exploring experimentally-determined PDB structures and Computed Structure Models at the RCSB Protein Data Bank (56).

Multiple glycosylation sites have been identified in MeV and MuV located at or near epitope binding sites suggesting that glycan shielding could play a role in MeV and MuV immune evasion (see **Figure 1**). This justifies the pursuit of similar correlative studies on glycosylation sites and antibody neutralization that have previously been conducted with other paramyxoviruses (42–44, 51).

### 2.1 MeV glycosylation sites and epitopes

The measles H protein has six known N-linked glycosylation sites that include Asn-168, Asn-187, Asn-200, Asn-215, Asn-238, and Asn-416 (see **Table 3**) (51, 57). To date, O-linked glycosylation sites have not been reported for MeV. The measles F protein has N-linked glycosylation sites on the F2 subunit peptide asparagine (Asn) residues 29, 61, and 67. Asn-29 and Asn-61 mutants decrease cell surface expression, and Asn-67 mutants reduce cell surface transport (see **Table 4**) (51). Still, the mechanisms by which this observed reduction in cell surface transport occurs is unknown.

**Table 3:** MeV H protein sequence alignment amino acid residue differences between the wild-type MeV genotype D3 strain and MeV vaccine strains.

Amino acid residue	Measles genotype D3	Measles vaccine
4	H (Histidine)	Q (Glutamine)
**141**	D (Aspartic acid)	N (Asparagine)
174	A (Alanine)	T (Threonine)
176	A (Alanine)	T (Threonine)
211	S (Serine)	G (Glycine)
235	G (Glycine)	E (Glutamic acid)
243	G (Glycine)	R (Arginine)
252	H (Histidine)	Y (Tyrosine)
276	F (Phenylalanine)	L (Leucine)
284	F (Phenylalanine)	L (Leucine)
295	R (Arginine)	K (Lysine)
296	F (Phenylalanine)	L (Leucine)
302	R (Arginine)	G (Glycine)
306	V (Valine)	I (Isoleucine)
308	V (Valine)	I (Isoleucine)
390	N (Asparagine)	I (Isoleucine)
**416**	N (Asparagine)	D (Aspartic acid)
446	T (Threonine)	S (Serine)
481	N (Asparagine)	Y (Tyrosine)

Bolded amino acids in the first column represent amino acid residues that are both located at identified sites of glycosylation as well as identified sites for an MeV antigenic epitope.

**Table 4:** MeV F protein sequence alignment amino acid residue differences between the wild-type MeV genotype D3 strain and MeV vaccine strains.

Amino acid residue	Measles genotype D3	Measles vaccine
2	D (Aspartic acid)	G (Glycine)
8	F (Phenylalanine)	S (Serine)
10	T (Threonine)	I (Isoleucine)
456	K (Lysine)	R (Arginine)
524	R (Arginine)	K (Lysine)

None of the mutated amino acids were located at both an identified site of glycosylation and an identified site for an MeV antigenic epitope.

The primary immunodominant epitope of MeV is located on the H protein near the receptor-binding site (RBS) (57). This region, also known as the hemagglutinating and noose epitope (HNE), is crucial for the MeV’s ability to bind to and infect host cells (57). The HNE region is highly conserved among different strains of MeV, contributing to the MeV’s antigenic stability (57). Located near the RBS is a neutralizing epitope that is shielded by an N-linked sugar in certain MeV mutants, including the Japanese H1 strain and the CAM-70 vaccine strain (57).

Glycosylation sites for MeV that overlap with MeV neutralizing epitopes include amino acids 141, 235, and 416 on the MeV H protein. Because both new glycosylation sites as well as sites important for epitope binding are being discovered for MeV, this is not an exhaustive list of the sites which overlap with each other. Overlapping glycosylation and epitope binding sites are of key interest since glycosylation of these sites will have a direct effect on the ability of the epitope to be recognized during infection. Thus, future studies involved in the identification of both sites are necessary to better understand how MeV evades immune recognition.

### 2.2 MuV glycosylation sites and epitopes

There are nine known N-linked sites of glycosylation on the mumps HN protein at amino acids 12–14, 127–129, 284–286, 329–331, 400–402, 448–450, 464–466, 507–509, and 514–516 (see **Table 5**) (58). Furthermore, these sites, though not yet identified as sites for glycosylation, are antigenic: 265–288, 329–340, and 352–360 (43). Along with GalNAc-modified trisaccharides containing α2,3-linked sialic acid, those which contained sialyl Lewis X (SLeX) were likewise identified as MuV binding motifs (44). Thus, SLeX is likely to aid receptor recognition by MuV. The SLeX is the terminal structure for some highly O-glycosylated mucins and sphingoglycolipids (44). Hence, while no defined sites of O-linked glycosylation have been identified for MuV as have been for MeV, SLeX may bind to such sites (44, 58). SLeX could be used in future studies to identify potential O-linked glycosylation sites for MuV. In addition, SLeX is relevant to study in MuV research because SLeX is broadly distributed throughout various tissues (59, 60). Thus, SLeX may be one defining factor in the establishment for widespread MuV dissemination within infected hosts (44, 58).

**Table 5:** MuV HN protein sequence alignment amino acid residue differences between the wild-type MuV genotype G strain and MuV vaccine strains.

Amino acid residue	Mumps genotype G	Mumps vaccine
6	F (Phenylalanine)	L (Leucine)
12	S (Serine)	N (Asparagine)
85	T (Threonine)	A (Alanine)
**113**	A (Alanine)	S (Serine)
**146**	R (Arginine)	K (Lysine)
375	I (Isoleucine)	V (Valine)
399	S (Serine)	N (Asparagine)
403	M (Methionine)	L (Leucine)
410	L (Leucine)	V (Valine)
440	S (Serine)	T (Threonine)
444	P (Proline)	Q (Glutamine)

Bolded amino acids in the first column represent amino acid residues that are both located at identified sites of glycosylation as well as identified sites for an MuV antigenic epitope.

MuV epitopes are still being mapped. However, one antigenic epitope is aa 329–340 on the HN protein (43, 58). One study expressed a fragment of the MuV hemagglutinin-neuraminidase protein HN3 (amino acids 213–372) in HeLa cells and used this purified fragment to immunize rabbits, generating anti-HN3 serum that could neutralize both attenuated and wild-type strains of MuV (61). This demonstrated that the HN3 region contains a key neutralizing epitope critical for immune protection.

Glycosylation sites for MuV that overlap with MuV neutralizing epitopes include amino acids 113, and 146 on the MuV HN protein and amino acids 16, 125, and 454 on the MuV F protein. Similarly, as for MeV, MuV glycosylation and epitope binding sites are still being mapped. For this reason, the list of overlapping glycosylation and epitope binding sites for MuV is expected to grow as more sites become identified. Such glycosylation and epitope binding site identification studies for the MeV and MuV surface glycoproteins MeV-H, MeV-F, MuV-HN, and MuV-F are thus needed in order to further our understanding of how MuV evades immune recognition.

### 2.3 Glycosylation sites for vaccine strains of Mev and MuV

The prior examples were relevant for wild-type strains of MeV and MuV. Vaccine strains of these viruses were attenuated through serial passaging in various non-human cell lines, a process which introduced numerous mutations and amino acid (aa) changes. This process will be described for each virus in more detail later on (62–74). These live-attenuated vaccine strains elicit a robust immune response that is sufficient for protecting against wild-type virus infections without inducing their corresponding disease and symptomatology (9, 14).

Because the live-attenuated MeV and MuV strains are mechanistically similar to the wild-type viruses, viral glycosylation may also impact their immunogenicity (62–74). This important question has not been adequately explored and will require research that distinguishes which aa sequence changes (if any) result in differential glycosylation patterns between the wild type and vaccine strains. The protein sequences of various MeV and MuV virus vaccine strains have been reported (62–74). We used these sequences to perform a protein-to-protein BLAST (blastp) for both the H protein in the wild type MeV with various MeV vaccine strains, and the HN protein in the wild type MuV with various MuV vaccine strains. Intriguingly, the BLAST comparison revealed that many of the amino acid substitutions were shared among multiple vaccine strains (see **Tables 3**-**6**). This holds true for both MeV and MuV (62–74).

**Table 6:** MuV F protein sequence alignment amino acid residue differences between the wild-type MuV genotype G strain and MuV vaccine strains.

Amino acid residue	Mumps genotype G	Mumps vaccine
15	P (Proline)	S (Serine)
16	F (Phenylalanine)	S (Serine)
**125**	I (Isoleucine)	V (Valine)
50	Y (Tyrosine)	I (Isoleucine)
170	N (Asparagine)	D (Aspartic acid)
330	N (Asparagine)	H (Histidine)
**454**	I (Isoleucine)	S (Serine)
479	I (Isoleucine)	V (Valine)
488	I (Isoleucine)	V (Valine)

Bolded amino acids in the first column represent amino acid residues that are both located at identified sites of glycosylation as well as identified sites for an MuV antigenic epitope.

MeV was first attenuated into the Edmonston A and Edmonston B strains (62). From Edmonston A originated the Schwarz vaccine strain and from Edmonston B originated the Edmonston-Zagreb and Moraten strains (62). Among the substitutions between wild-type and live-attenuated MeV was a change in amino acid 416 of the H protein from N to D, which is a glycosylation site near immunodominant epitopes (57, 62–74).

MuV was first attenuated into the Jerryl Lynn strain. Two lineages were later defined: Jerryl Lynn 2 (JL-2) and Jerryl Lynn 5 (JL-5), which exist in different proportions in the current MMR-II trivalent vaccines distributed in the US and Europe (63, 70–74). In the wild type MuV, additional glycosylation sites can be found at HN amino acid residues 284 (asparagine) and 329 (asparagine) in MuV vaccine strains (44, 50). The amino acids at residues 284 and 329 in the HN protein are the same between wild type and vaccine strain mumps viruses (70–74).

Differences in amino acids at sites of glycosylation between the wild type and vaccine strain of each virus could potentially change the way each are glycosylated. This signifies that immune recognition and evasion differences between the wild type and live attenuated viruses may be the result of how each are glycosylated during replication. More research on the effects of modifications to amino acid residues of viral glycosylation sites on glycosylation between wild type and vaccine strains of measles and mumps will be needed before immune responses differences between the wild type and live attenuated viruses can be attributed to glycosylation.

An increased understanding of how amino acid changes alter glycosylation and subsequently change the immunogenicity of these viral strains, could potentially lead to the design of improved vaccine strains that elicit enhanced immunity (75, 76). Early promises have already been shown in this field with the novel design of a mumps vaccine using recombinant surface glycoproteins MuV-F and MuV-F/HN (77). In brief, Loomis et al. generated purified recombinant prefusion-stabilized MuV-F (pre-F) and recombinant prefusion-stabilized MuV-F/HN glycoproteins (pre-F/HN) and then compared neutralizing antibody responses between the two in a mouse model (77). The Pre-F/HN immunization demonstrated a better neutralizing activity elicited through the presentation of a greater diversity of antigenic sites (77). A similar method could be employed to produce recombinant mumps and recombinant measles vaccines with carefully controlled glycosylation patterns to shape the resulting immune response and avoid glycan shielding (77). These new vaccines would thus contain live-attenuated virus strains that are disarmed of one of their most fine-tuned “weapons” against host immunity. Another potential enhancement would be the inclusion of glycosylation inhibitors within vaccine doses to suppress the ability of the vaccine strains to become shielded by host glycans factors to begin with (77, 78). Hence, these next generation vaccines would provide major advantages.

The first live-attenuated vaccines to be generated for MeV and MuV were both taken from an infection with the naturally occurring wild-type virus (62–74). However, there is some implication that mumps vaccine strains may be more virulent as compared to measles vaccine strains. For example, there have been previous reports of horizontal transfer of mumps vaccine virus between a vaccine recipient and uninfected and unvaccinated individuals (79–81). One such instance occurred in western Suriname in South America. Here, an increase in mumps cases within the region were reported after a mass immunization campaign and two school immunization campaigns took place, with a mumps attack rate of 15.1% in immunized children and an attack rate of only 4.7% in those who were not immunized (63). This study could not distinguish the underlying cause, which may have been the baseline virulence of the Leningrad-Zagreb mumps strain or production errors for the specific lots of the vaccine that were administered to this population (63). In Novosibirsk, western Siberia, Russia, an outbreak of 216 cases of mumps emerged where approximately 75% of reported cases had previously received at least one dose of the Leningrad-3 live-attenuated vaccine where observed clustering among vaccinees further supported that this likely resulted as a failure of vaccine-induced immunity under outbreak exposure (79, 80). The molecular testing from another study demonstrated that the Novosibirsk outbreak strain of mumps was genetically distinct from the vaccine strain, strengthening the case that vaccine−induced immunity was insufficient against this wild−type strain under these conditions (79–81). Additionally, a study of mumps cases occurring between 2010 and 2011 in Minsk, Belarus reported symptomatic transmission of the L-Zagreb vaccine strain of mumps, confirmed by viral sequencing (81). In contrast, the MeV live-attenuated vaccines have never been correlated with the occurrence of viral outbreaks amongst populations post-immunization.

## 3 Glycosylation shields viral innate immune activation

**Figure 2:**
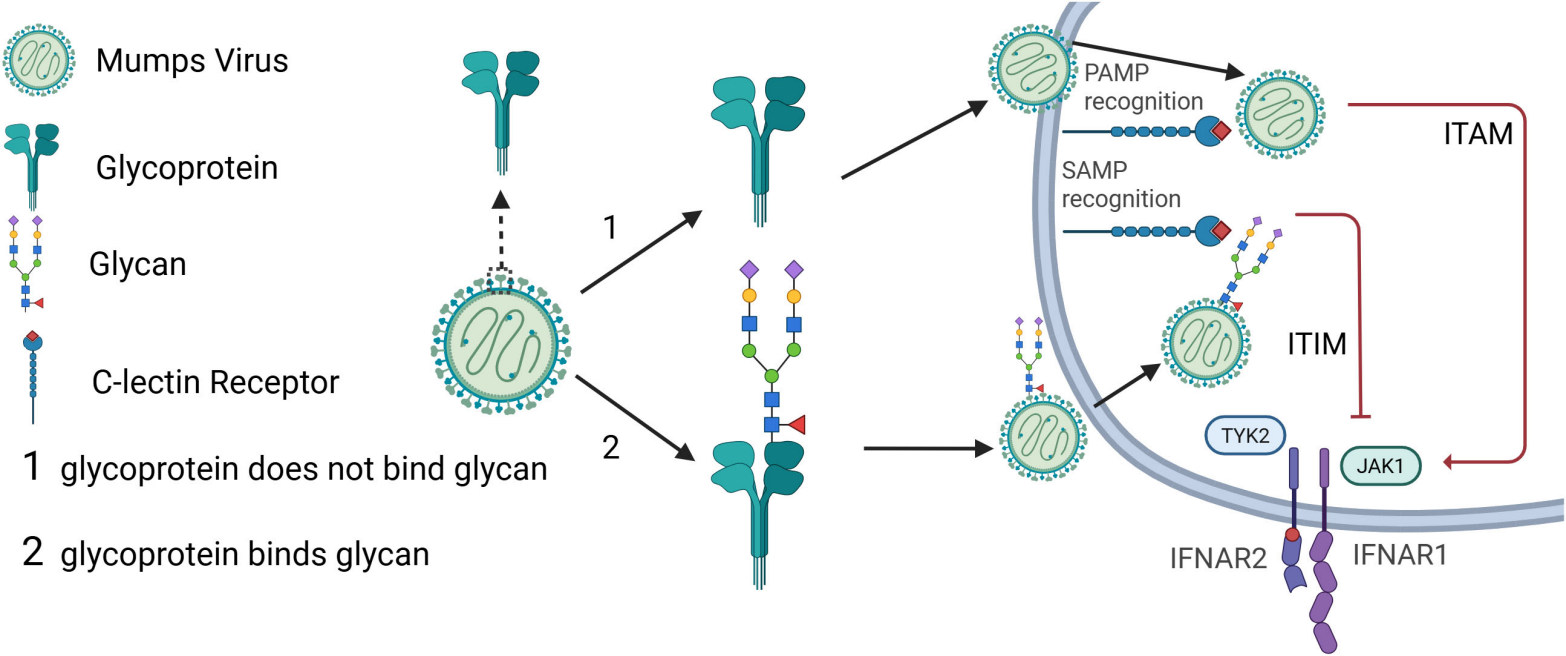
Graphical abstract. The surface glycoproteins of measles or mumps virus will either (scenario 1) not become glycosylated by a host glycan or (scenario 2) become glycosylated by a host glycan. For scenario 1, the MeV or MuV surface glycoproteins (MeV-F, MeV-H, MuV-F, or MuV-HN) will be recognized by a c-lectin receptor as a PAMP and activate the type I IFN pathway which leads to the transcription of antiviral ISGs that further modulate downstream immune responses. For scenario 2, the MeV or MuV surface glycoprotein will be recognized by a c-lectin receptor as a SAMP and inhibit the type I IFN response along with preceding downstream immune responses.

The glycans on MeV and MuV envelope proteins can hide MeV and MuV antigenic epitopes from recognition by circulating Abs or pattern-recognition receptors (PRRs), a process that we have defined here as glycan shielding (see **Figure 2**). Glycans can exhibit another important immunomodulatory effect through by acting as host innate immune suppression factors called self-activating molecular patterns (SAMPs) (82). When a SAMP is recognized instead of a pathogen-associated molecular pattern (PAMP) by toll-like receptors (TLRs), the complement system will be deactivated to preserve what the system perceives to be a healthy tissue (82, 83). Specifically, SAMP recognition will inhibit the IFN 1 innate immune response by blocking TYK2 and JAK1 association with IFNAR1/2 (83, 84).

### 3.1 PAMP recognition by CLRs

PAMPs include viral nucleic acids, proteins, sugars, and lipids that are recognized by host cell PRRs thereby inducing various innate immune responses to the pathogen (85). One example of immune activation after PAMP recognition by a PRR is genomic viral RNA binding to TLR-7/8, resulting in type I and type III interferon responses (86). Another example is the c-lectin receptor (CLR), a PRR that recognize PAMPS composed specifically of carbohydrates, which modulates the complement system as well as inflammatory and innate immune pathways such as NFkB, and secretion of type I Interferons (IFN I) (87–94).

CLR recognition can induce signaling cascades that lead to the activation of nuclear factor kappaB (NF-κB) transcription factors via Syk- and CARD9-dependent pathways (88). The NF-κB pathway is important for stimulating early innate and inflammatory host responses to viral infections (88). However, small sugar-associated molecules are able to successfully inhibit this pathway (95). Two examples of these are engeletin and Gal-3BP. Engeletin, a glycoside, has shown promise in inhibiting the pathway in inflammatory and autoimmune diseases (91, 92). Gal-3BP serves as a negative regulator of the NF-κB pathway by inhibiting TAK1 activation (91). Gal-1 (C7.3) has also been demonstrated to attenuate NF-κB responses (92).

### 3.2 SAMP recognition by CLRs

SAMPs are nucleic acids, proteins, sugars, and lipids that are native to the host (83). SAMPs are recognized by PRRs and suppress their activation, thereby inhibiting immune responses (83). SAMPS can also be recognized by CLRs. For instance, one CLR is the dendritic cell immunoreceptor (DCIR) which recognizes human Lewis b antigen-a blood group carbohydrate epitope (83, 96). Recognition by DCIR by this SAMP will result in the activation of the DCIR-specific ITIM to maintain immune homeostasis (96). Glycosylation of viral capsid proteins by host factors may allow for the viral protein PAMPs to be covered by host glycan SAMPS and be recognized as such by host cells (82). This means that once viruses become glycosylated, host glycan binding proteins such as galactins, siglecs, and c-type lectins would recognize the SAMP on the virus and activate signaling pathways that can modulate and inhibit the innate immune response (83, 91–94). Therefore, SAMP recognition in place of PAMP recognition will inactivate the complement system, suppressing innate immune responses (82, 83). For this reason, the term glycan shielding originally used to describe blocking antibody binding to viral proteins should be expanded to include suppression of innate immune responses, thus ‘shielding’ the pathogen from the two major arms of the immune system. This makes glycan shielding a convenient way for MeV and MuV to both infect host cells in spite of host humoral immune responses and remain unnoticed by innate immune responses within the cell long enough for efficient replication.

MeV and MuV share the immune evasion strategy of suppressing the type I IFN response (97, 98). One way this is achieved is via STAT1 and STAT3 inhibition (21, 24). IFN type I responses can also be suppressed by the recognition of glycans, as has been shown as a result of BDCA-2 (a glycan-binding receptor) glycan recognition (99, 100). Siglec-G, for example, can become induced by RNA viruses to degrade RIG-I and suppress the IFN I pathway to allow for viral immune evasion (101).

## 4 Translational implications for MeV and MuV glycosylation

### 4.1 Glycosylation inhibitors

Studying the immunogenicity of measles and mumps glycosylation can have profound translational impacts in healthcare. For instance, glycosylation inhibitors may have a beneficial effect on the immune response to viruses while simultaneously inhibiting viral infection and spread within a host. The potential anti-viral effects of these inhibitors should be explored with an eye toward safety and balancing positive effects with any side effects. Glycosylation inhibitors have been considered for their use as an antiviral for SARS-CoV-2 infection (102). However, they are not an approved antiviral drug. Yet, there is promise for their future use as antivirals given that following glycosylation inhibitors have already been FDA-approved for other indications (103, 104). For instance, acarbose and miglitol are used to treat type II diabetes, and their potential role as RNA virus antivirals have been implicated (103). Miglustat is approved for treating Gaucher’s disease (104). In addition to these, two other drugs have been approved for treating type II diabetes, metformin and statins, which previously corresponded with higher galactosylation and sialylation in glycans and lower fucosylation (105). All of these are FDA approved glycosylation inhibitors that have already been widely used among patients, so the implementation of their indication for use as antiviral and immunosuppressant treatments in clinical trials is within the framework of medical ethics. Further research into the use of these and other glycosylation inhibitors as antiviral drugs, especially for the treatment and/or prevention of measles and mumps, has the potential to arm health professionals with a useful tool to counter a measles and/or mumps outbreak should one occur.

### 4.2 Glycosylation enhancers

Just as inhibitors of viral glycosylation have the potential for use as antivirals, on the opposite side of the same coin, glycosylation enhancers could be used to treat certain autoimmune diseases and cancers. MuV and MeV have separately had success as a therapy to target and kill cancer cells (106–108). Moreover, cancer cells that received the highest viral load had the lowest activation of IFN (106–108). If glycosylation sites are able to aid MeV and MuV to enter cells, glycosylation enhancers could potentially allow more oncolytic MeV and MuV to reach cancer cells during treatment. For instance, it is likely that many cancer patients have been either immunized against or been naturally infected by MeV and MuV. Therefore, if either virus is used as an oncolytic therapy, it would require evading neutralization by pre-existing host Abs (43–55, 57–61). However, adding glycans to MeV and MuV oncolytic viruses could potentially help them reach their target cells before becoming neutralized. Therefore, in the case of cancer therapy, patients might benefit from the addition of glycans to these viruses to increase the efficacy of their therapy (109–111). MeV and MuV glycosylation also contain the properties of being able to modulate the innate immune response of the host cell currently infected by serving as a SAMP that diminishes immune stimulation (82, 83). Enhancing the glycosylation of oncolytic MeV and MuV during cancer therapy would, thus, not only allow for more cancer cells to become infected with MeV and MuV but may facilitate increased infection and replication (109–111). Thus, glycosylation enhancers could increase the potency of MeV and MuV oncolytic therapies. In addition, specific epitope fragments of MeV and MuV could be given in a cocktail with glycosylation enhancers to trigger SAMP recognition for treatment of various autoimmune or inflammatory diseases.

## 5 Conclusion

Measles and mumps viruses both undergo glycosylation during their infectious cycle. While it is known that other paramyxoviruses such as HiV and HeV use glycosylation as a strategy to evade neutralization and recognition by host antibodies, it remains unclear what the effects of glycosylation on the immune responses generated to measles and mumps are. However, a protein sequence alignment analysis showed that several amino acid site changes occurred in the attenuation of the wild type measles virus strain to different live-attenuated vaccine strains of measles virus. This was observed for mumps as well. If viral glycosylation affects host immune responses to measles and mumps in the same way that glycosylation of other paramyxoviruses does, differences in glycosylation patterns between vaccine strains may alter the effectiveness of each vaccine component and their ability to induce long-lasting immunity. To prevent disease outbreaks from occurring in the current environment where waning immunity is increasing, there is a need for more in-depth studies of the pathogenic and immunogenic properties of live attenuated MeV and MuV. One study should focus on how amino acid site mutations such as those which occurred between wild type and live attenuated strains of MeV and MuV can impact viral glycosylation.

MeV and MuV glycosylation research also holds potential for use in various antiviral and immunotherapeutics. The ability for host glycans to be recognized by CLRs as SAMPs and activate ITIMs insinuates probable cause for certain glycosylation patterns of MeV and MuV to do the same. This would allow for MeV and MuV bearing these specific glycosylation patterns to modulate immune responses in the virus’ favor so that type I IFN and early antiviral responses are inhibited.

Since glycosylation can be used by viruses as an effective strategy to evade the immune system, glycosylation inhibitors could be used as antivirals as well as a therapeutic for certain chronic immunodeficiency diseases such as HIV/AIDS (112, 113). Alternately, drugs that can alter glycosylation will be useful to increase cancer cell death during oncolytic MuV and MeV therapy (114, 115). Glycan modulators in combination with MuV and MeV fragments that can become glycosylated could also be used to treat autoimmune and inflammatory diseases (116, 117). There is a long way before any of these therapeutics might become translated. However, more research that leads to a comprehensive understanding of the properties of MeV and MuV glycosylation patterns and their effects on immune recognition is the first step. A future study should entail the identification of immune responses to glycosylation site changes to MeV and MuV to gain novel insight into the immunogenic potential of MeV and MuV glycosylation.
